# Transplantation of the Ovary to Avoid Dysmenorrhea and Premature Menopause*Read before the joint meeting of Caldwell, Gonzales and Guadalupe Medical Societies, Luling, Texas, July 14, 1914.

**Published:** 1914-07

**Authors:** Z. T. Scott

**Affiliations:** Surgeon to Austin Presbyterian Sanitarium


### TEXAS MEDICAL JOURNAL

Dr. F. E. Daniel, Founder. Established July, 1885

MRS. F. E. DANIEL, - Publisher and Managing Editor

Published Monthly.—Subscription, $1.00 a Year.

VOL. XXX AUSTIN, JULY, 1914 NO. 1

The publisher is not responsible for views of contributors

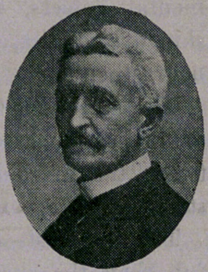

For Texas Medical Journal.

### Transplantation of the Ovary to Avoid Dysmenorrhea and Premature Menopause.*

BY Z. T. SCOTT, M. D., SURGEON TO AUSTIN PRESBYTERIAN SANITARIUM.

The last decade has brought forth much comment and investigation by physiologists, anatomists, physicians and surgeons in the field of internal secretions. Possibly there is no department of medicine which offers greater opportunity for research than this.

Biedl, Falta, Sajous and Gilford have elaborated upon the theories and experiments set forth by Brown, Sequard, Johannes, Miller and Claud Bernard, while the late Roswell Park has clearly reviewed our present knowledge of ductless glands and their hormones in his paper before the Clinical Congress of Surgeons of North America in 1913.

That the secretions of these glands undoubtedly exercise a control of many of our vital processes, witness the influence of the ovary in menstruation, the thyroid in cretinism, the thymus in bone development, and the testis in the development of the male.

Those ductless glands which are now recognized as such are as follows: (1) Hypophysis: (a) anterior; (b) posterior. (2) Epiphysis or pinial body. (3) Thyroid. (4) Parathyroids. (5) Thymus. (6) Adrenals and (7) Chromaffin system. (8) Pancreas: Intertubular cell groups; Island of Langerhans. (9) Genital glands: Ovaries, testes.

A partial list of those diseases which are more or less directly due to a modified secretion of these glands are as follows: Acromegaly; hydrocephalus; Graves' disease; myxedema and cretinism;

*Read before the joint meeting of Caldwell, Gonzales and Guadalupe Medical Societies, Luling, Texas, July 14, 1914.

tetany; thymic asthma and status lymphaticus; rickets; obesity; Addison's disease; sexual changes—amenorrhea and eunuchism; mental defects, infantilism, mongolism and mental precocity; skin dystrophies, dryness, excessive perspiration and loss of hair; neurasthenia.

The secretions or hormones of these glands may be individual or intimately associated with others; e. g., the relationship between the thyroid, the ovary and the mammary gland in menstruation; the pancreas and hypophysis in diabetes.

The internists have been more aggressive, if not more successful, than the surgeons in their remedies for the pervertions of internal secretions and many of these agents are familiar to us all; e. g., adrenalin, extracts of the thyroid, thymus, ovary, pituitary and others. However, the surgeons are close upon them in their work of organic-excision for hyper-secretion and organo-transplantation for hypo-secretion. There are few surgeons who have not seen the beneficial results of thyroidectomy in hyperthryoidism, on the one hand, and the cessation of the various nervous phenomena associated with premature menopause following transplantation of the ovary. Personally, we have had the most satisfactory results in transplantation of the ovary, especially where it was necessary to remove both glands from their normal position for degeneration or for widespread adhesions. All surgeons have long sought to preserve these glands intact, but frequently it becomes imperative to remove them. In this class of cases, we prefer to implant a portion or all of one ovary between the rectus and peritoneum, and in no case have we had cause for regret.

The relation of the ductless glands to surgery has been well set forth in the classification of park as follows:

Conditions Which May Require Surgical Intervention.—Thyroid: Extirpation, which must be partial, save when dealing with malignant disease; ligation of blood vessels; transplantation or grafting. Parathyroids: Here we can scarcely do more than transplanting. Thymus: Extirpation or transplantation. Adrenals: Transplantation, or possibly extirpation of one. Cachexia ovari priva: Has been relieved by transplantation. Hypophysis: The anterior lobe may be incompletely removed; very rarely is complete extirpation practicable; transplantation has been attempted, so far with questionable success. Genital glands: May be removed for various causes, but removal is rarely demanded for conditions under present consideration. Breasts and tonsils: Removal.

Operations Which May Be Required.—In general, the character of the operations called for under the various conditions above mentioned include: Transplantation (thyroid, thymus, adrenal, genital); craniectomy and decompression, for consequences of actual synotosis, and consequent and microcephaly, etc.; operations for hydrocephalus; operations on the spine and limbs for various deformities; thyroidectomy and ligation of thyroidal vessels; thymectomy and operations required by pressure upon the trachea (tracheotomy); operations for consequences of osteomalacia, achondroplasia, etc., involving the pelvis (Caesarean section, etc.); operations on the teeth and jaws for various congenital defects, cleft palate, etc.

The technic for transplantation of tissue is simple, and requires no unusual degree of asepsis, the only prerequisite being the acccss to a liberal blood supply. We endeavor to tack the hilum of the ovary between the fibers of the rectus muscle with a single stitch of catgut, thereby inviting a liberal blood supply. In one of our cases, the ovary to be transplanted was removed and remained in warm normal saline solution for some ten or fifteen minutes while the broad ligament was repaired. This in no way affected the viability of the tissue. The only difficulty is in the fact that up to this time comparatively few cases have been explored later to inform us whether or not the tissue had been absorbed.

